# Reversal of Chloroquine Resistance in *Plasmodium falciparum* in Gabon: A Phenotype-Genotype Relationship over the Last 20 Years

**DOI:** 10.3390/ijms27083566

**Published:** 2026-04-16

**Authors:** Juliana Inoue, Miriam Rodi, Francis Emmanuel Towanou Bohissou, Lais Pessanha de Carvalho, Katharina Lohse, Erik Koehne, Selidji T. Agnandji, Ghyslain Mombo-Ngoma, Peter G. Kremsner, Andrea Kreidenweiss, Jana Held

**Affiliations:** 1 Institute of Tropical Medicine Tübingen, University Hospital Tübingen, Wilhelmstraße 27, 72074 Tübingen, Germany; juliana.inoue@uni-tuebingen.de (J.I.); bohissouf@yahoo.fr (F.E.T.B.); lais.pessanha-de-carvalho@uni-tuebingen.de (L.P.d.C.);; 2 Centre de Recherche Entomologique de Cotonou, Cotonou 06 BP 2604, Benin; 3 Centre de Recherches Médicales de Lambaréné (CERMEL), Lambaréné BP 242, Gabon; 4 Institute for Medical Microbiology, University of Münster, 48149 Münster, Germany; 5 Research Group Drug Implementation, Bernhard Nocht Institute for Tropical Medicine & I. Department of Medicine, University Medical Center Hamburg-Eppendorf, 20246 Hamburg, Germany; 6 German Center for Infection Research (DZIF), Partner Site Tübingen, 72074 Tübingen, Germany

**Keywords:** chloroquine resistance, *Plasmodium falciparum*, *pfcrt* gene, *pfmdr1* gene

## Abstract

The emergence of chloroquine-resistant *Plasmodium falciparum* in the 1950s posed a global threat to malaria control. Ceasing chloroquine use restored chloroquine-sensitive strains in many African countries. To assess whether chloroquine sensitivity re-emerged in Lambaréné, Gabon, we compiled published and new data on *P. falciparum* ex vivo chloroquine susceptibility, quantified as half-maximal inhibitory concentration (IC_50_), from four time points between 2004 and 2024. We then assessed the prevalence of *pfcrt* and *pfmdr1* polymorphisms associated with chloroquine resistance via real-time PCR and Sanger sequencing, respectively, at six different time points between 2009 and 2024. Ex vivo chloroquine susceptibility data revealed a stepwise decrease in the median chloroquine IC_50_ from 2004 (113.8 nM; IQR: 79.6–163.5 nM, *n* = 42), 2009 (46.7 nM; IQR: 27.4–76.9 nM, *n* = 26), and 2017–2018 (15.6 nM; IQR: 6.1–40.9 nM, *n* = 46) to 2024 (2.1 nM; IQR: 0.8–6.1 nM, *n* = 39). The chloroquine-sensitive *pfcrt* haplotype CVMNK increased from 3.6% (1/28) in 2009 to 98.2% (56/57) in 2024, as well as the wild-type *pfmdr1* N86 (23.1% (6/16), 2009; 100% (19/19), 2024). Paired molecular and ex vivo analyses revealed an association between IC_50_ values and CVMNK *pfcrt* and *pfmdr1* N86. Our data provide evidence for the reestablishment of chloroquine-sensitive *P. falciparum* in Lambaréné, Gabon.

## 1. Introduction

In 2024, *Plasmodium falciparum* malaria caused an estimated 282 million cases and 610,000 deaths worldwide [1]. Prompt diagnosis and effective treatment remain essential for malaria control. However, the emergence of drug-resistant *P. falciparum* strains threatens treatment efficacy. For decades, chloroquine (CQ), a 4-aminoquinoline compound, was the first-line antimalarial therapy. Its widespread use contributed to significant reductions in the worldwide malaria burden until the emergence of CQ-resistant parasites. Resistance was first reported in the late 1950s, emerging independently in Southeast Asia, South America, and Papua New Guinea and subsequently spreading to multiple regions, including Africa [2,3]. In East Africa, CQ-resistant *P. falciparum* was first documented in 1979 and within a decade spread to West Africa [4,5,6,7]. In Lambaréné, Gabon, clinical trials conducted in the early 1990s confirmed a high level of CQ resistance, shifting Albert Schweizer Hospital’s treatment policy to sulfadoxine-pyrimethamine [8,9,10]. In 2003, following the World Health Organization (WHO) guidelines, Gabon adopted artemisinin-based combination therapies (ACT)—artemether-lumefantrine and artesunate-amodiaquine—as first-line treatment for malaria [11].

Chloroquine resistance in *P. falciparum* is associated with single-nucleotide polymorphisms (SNPs) in the *P. falciparum* chloroquine resistance transporter (*pfcrt*) gene, which encodes a transmembrane protein located in the parasite’s digestive vacuole [12]. CQ-resistant clinical isolates harbor a lysine-to-threonine substitution at codon 76, while laboratory-adapted lines can also present asparagine or isoleucine at this position [13]. The CV**IET** haplotype, defined by amino acid substitutions at codons 72–76 of *pfcrt*, is predominant in Africa. Whereas the **S**VMN**T** haplotype, also associated with amodiaquine resistance [14], is more frequent in South America, Papua New Guinea, the Philippines, and Indonesia [15,16,17]. Other studies provided evidence that *P. falciparum* multidrug resistance 1 (*pfmdr1*) mutations may modulate in vitro CQ resistance in parasites exhibiting *pfcrt*-mediated resistance by decreasing CQ accumulation in vitro [18] and reported linkage disequilibrium between *pfcrt* 76**T** and *pfmdr1* 86**Y**, indicating drug-driven co-transmission [19]. Various codons of *pfmdr1* seem to influence *P. falciparum* susceptibility to several antimalarials, including CQ and amodiaquine [20,21].

Mutations in the *pfcrt* gene carry a high fitness cost for the parasite [22], and CQ withdrawal from treatment guidelines allowed re-emergence of sensitive strains in several African countries. First reported in Malawi, *pfcrt*-mutant parasites dropped from 85% in 1992 to 13% in 2000, seven years after sulfadoxine-pyrimethamine replaced CQ [23,24]. This was also confirmed in vitro and clinically in 2001, when CQ cleared 100% of 63 uncomplicated *P. falciparum* infections in Malawi [24]. In Kenya, CQ withdrawal from treatment guidelines occurred in the late 1990s, and a significant increase in wild-type *pfcrt* proportion was reported, from 61.2% (2010) to 93.0% (2013) [25]. In Zambia and Uganda, *pfcrt*-mutant parasites were no longer detected following 10 and 11 years, respectively, after changes in treatment policy [26,27].

In Lambaréné and surrounding villages in Gabon, CQ in vitro resistance was first reported in 1984, with 19% of isolates exhibiting a resistant profile [28]. Subsequent studies in the early 1990s documented a sharp increase, with resistance reaching 94–100% [10,29]. Lower resistance rates were observed in the following years, 55% and 73% in 1996 [30,31] and 45% in 1998 [32]. These findings contrasted with molecular data from 1996, which identified the *pfcrt* 76**T** mutant allele in 100% of tested isolates, retrospectively [33]. In 2001, a reassessment confirmed high-grade in vivo and in vitro resistance [34], and molecular analyses from 2001 and 2002 cohorts showed a 100% prevalence of the 76**T** mutation [33,35]. An assessment conducted between two and four years after ACTs replaced CQ revealed a slight decrease in the prevalence of mutant *pfcrt* to 97% among *P. falciparum* collected between 2005–2007 [36]. Further studies with samples from 2016 [37] and 2018 [38], 13 and 15 years after CQ withdrawal, reported 89% and 78.5% prevalence of mutant parasites, respectively.

To assess whether CQ sensitivity has re-emerged in Lambaréné, Gabon, we gathered ex vivo CQ-susceptibility data on *P. falciparum* isolates collected in 2004, 2009 and 2017–2018 from previously published studies and newly assessed data from 2024 from this area. Additionally, codons 72–76 of *pfcrt* and 86, 184, 1034, and 1246 of *pfmdr1*, associated with CQ response, were genotyped.

## 2. Results

### 2.1. In Vitro Reversal of Chloroquine Resistance in Plasmodium falciparum Parasites

**Figure 1:**
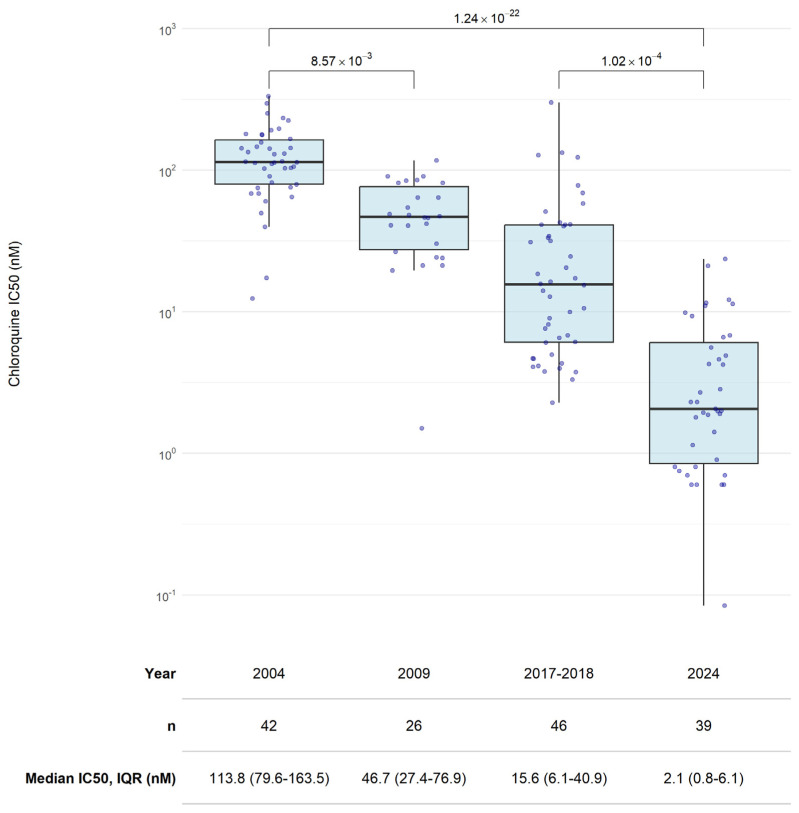
Chloroquine (CQ) susceptibility of *P. falciparum* isolates from Lambaréné and surrounding villages in Gabon over the years after CQ was officially removed from national treatment guidelines in 2003. The upper panel displays CQ IC_50_s (nM) across the sampled years. Each dot represents one isolate tested in duplicate. The bottom table summarizes the sample size (*n*) and the median IC_50_s and IQR (nM) for each year. Previously published data: 2004 [41], 2009 [39], and 2017–2018 [40].

Altogether, CQ ex vivo data were obtained from 153 isolates from four time points. Figure 1 shows a stepwise decrease in half-maximal inhibitory concentration (IC_50_) values throughout the years and a significant decrease in the median IC_50_ from 113.8 nM (IQR 79.6–163.5 nM) in 2004 to 2.1 nM (IQR 0.8–6.1 nM) in 2024. Data from isolates collected in 2004, 2009, and 2017–2018 were previously published [39,40,41].

### 2.2. pfcrt Haplotypes Prevalence over 20 Years

**Figure 2:**
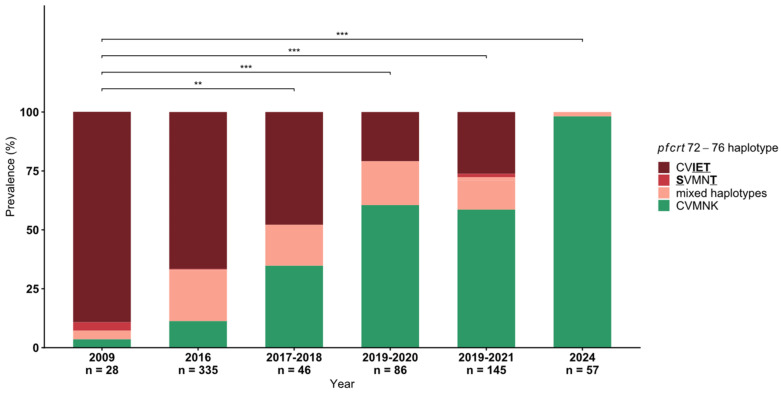
Prevalence of *pfcrt* codons 72–76 haplotypes from 2009 to 2024 in *Plasmodium falciparum* isolates from Lambaréné and surrounding villages in Gabon, showing a decrease in the prevalence of the CQ-resistant CV**IET** haplotype (dark red) and an increase in the CQ-sensitive CVMNK haplotype (green) throughout the years (** = *p* < 0.01, *** = *p* < 0.001, Pearson’s Chi-squared test). Mixed haplotypes are those with two or three haplotypes detected in the same sample. Mutant amino acids are bold and underlined. Data from 2016 has been previously published [37].

Genotyping of *pfcrt* codons 72–76 was performed on samples from 2009 (*n* = 28), 2016 (*n* = 335), 2017–2018 (*n* = 46), 2019–2020 (*n* = 86), 2020–2021 (*n* = 145), and 2024 (*n* = 57), totalizing 697 samples. The prevalence of single infections with the sensitive haplotype, CVMNK, consistently increased over time from 3.6% (1/28) in 2009 to 98.2% (56/57) in 2024 (Figure 2). Table 1 shows the detailed prevalence of *pfcrt* haplotypes per year. Isolates with mixed haplotypes CVMNK/CV**IET** were present at 20.9%, 17.4%, 18.6%, and 12.4% in 2016, 2017–2018, 2019–2020, and 2019–2021, respectively. CVMNK/**S**VMN**T** were identified in 3.6%, 0.6%, 0.7%, and 1.8% of the isolates in 2009, 2016, 2019–2021, and 2024, respectively. CV**IET**/**S**VMN**T** and CVMNK/CV**IET**/**S**VMN**T** were identified in one isolate from 2019–2021 (0.7%) and 2016 (0.3%), respectively. The **S**VMN**T** haplotype was observed in 3.6% (1/28), 0.3% (1/335), and 1.4% (2/145) of isolates from 2009, 2016, and 2019–2021, respectively. Remarkably, the resistant haplotype CV**IET** was not found in isolates from 2024.

### 2.3. Prevalence of pfmdr1 Polymorphisms

Since *pfmdr1* mutations can modulate CQ resistance [18] in *pfcrt*-mutant parasites, we genotyped codons 86, 184, 1034, 1042, and 1246 of *pfmdr1* in isolates with ex vivo and *pfcrt* genotyping data (2009, *n* = 26; 2017–2018, *n* = 43; 2024, *n* = 18) to further explore their contribution to CQ response. One sample from 2017–2018 failed to amplify. Table 2 shows a decrease in the prevalence of the mutant 86**Y** throughout the years, with 76.9% (20/26), 18.6% (8/43), and none of the genotyped samples harboring the mutation in single or mixed infections in 2009, 2017–2018, and 2024, respectively. Likewise, the mutant *pfmdr1* 184**F** decreased over time from 76.9% (20/26) in 2009 to 60.5% (26/43) in 2017/2018 and 52.6% (8/18) in 2024. Mutations at codons 1034, 1042, and 1246 were absent or in low frequency (2.3% (1/43) of 1246**Y** in isolates from 2017–2018).

### 2.4. Correlation of Ex Vivo Data and pfcrt and pfmdr1 Polymorphisms

**Figure 3:**
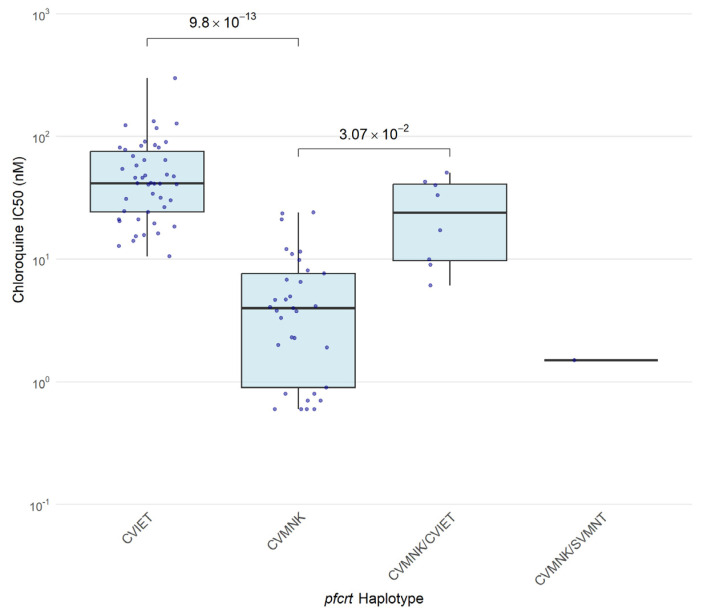
Association between *pfcrt* 72–76 haplotypes and chloroquine ex vivo susceptibility in *P. falciparum* collected in 2009 (*n* = 26), 2017–2018 (*n* = 44), and 2024 (*n* = 18). Box plots show the median IC_50_ (nM) and interquartile range. CVMNK: wild type; C**VIET** and **S**VMN**T**: mutant.

Next, we assessed the association of CQ ex vivo response and *pfcrt* polymorphisms. *Pfcrt* genotyping and ex vivo paired data were obtained from 88 isolates in total, from 2009 (*n* = 26), 2017–2018 (*n* = 44), and 2024 (*n* = 18). One sample from 2024 failed to amplify. Figure 3 shows the distribution of IC_50_ values according to the *pfcrt* haplotype. CQ median IC_50_ values of isolates harboring the *pfcrt* sensitive haplotype CVMNK only (*n* = 33) were 3.97 nM (IQR: 0.9–7.6 nM), in contrast to 41.61 nM (IQR: 24.2–75.6 nM) in isolates harboring the *pfcrt* resistant haplotype CV**IET** (*n* = 46) only. Isolates with mixed haplotypes CVMNK/CV**IET** (*n* = 8) had median IC_50_ values of 25.1 nM (IQR: 9.7–40.7 nM), and the only isolate with mixed CVMNK/**S**VMN**T** genotypes presented an IC_50_ < 1.5 nM.

Given the *pfmdr1* SNPs’ influence on CQ response, we subsequently analyzed the effect of multiple *pfmdr1* codons on CQ IC_50_s. In our dataset, only *pfmdr1* 86**Y** was associated with high IC_50_s (*p* = 0.00127). Combined *pfmdr1* 86 and *pfcrt* haplotypes data showed a dominant parasite population characterized by the haplotype CV**IET** 86**Y** (76.9%, 20/26) in 2009 (Table 2). The period of 2017–2018 seems a transitional phase with a heterogeneous parasite population displaying a wide range of CQ IC_50_ values and a higher genetic diversity compared to the other study periods, with isolates harboring the haplotypes CVMNK 86N (27.9%, 12/43), CVMNK 86**Y** (4.7%, 2/43), CV**IET** 86N (58.1%, 25/43), and CV**IET** 86**Y** (9.3%, 4/43). The present population (year 2024) comprises only parasites with the CVMNK 86N haplotype and a median IC_50_ of 2.1 nM for CQ.

### 2.5. Prevalence of pfcrt 76**T** in Gabon Compared to Published Data from Neighboring Countries

**Figure 4:**
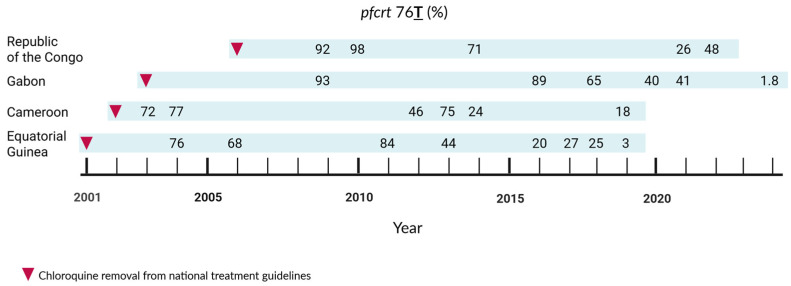
Spatiotemporal distribution of *pfcrt* 76**T** allele frequency. Temporal trends of *pfcrt* 76**T** in Gabon and the neighboring countries, the Republic of the Congo, Cameroon, and Equatorial Guinea, extracted from previously published data, after removal of CQ from the national treatment guidelines. Numbers indicate the percentage of resistant (*pfcrt* 76**T**) genotypes found in the respective studies. The study’s references are listed in the Supplementary Table S1. Created with BioRender. Held, J. (2026) https://BioRender.com/xnq75k7 (accessed on 8 April 2026).

We compared the temporal trends of the *pfcrt* 76**T** prevalence in Gabon with published data from its neighboring countries, the Republic of the Congo, Cameroon, and Equatorial Guinea (Supplementary Table S1), after CQ was officially replaced in the national treatment guidelines. Figure 4 shows a decline in the mutant allele across the four countries. While Gabon and Equatorial Guinea have seen resistance nearly disappear, declining to 1.8% and 3%, respectively, the Republic of the Congo shows a fluctuating but overall declining trend, with the most recent data available from 2022. In Cameroon, a decrease in the prevalence of mutant parasites was also observed in Yaoundé and in the coastal region (7% and 3%, respectively), 17 years after CQ discontinuation in 2019. In the northern region, 63% of isolates presented the mutation, setting the country’s overall prevalence to 18% during this period.

## 3. Discussion

Our findings provide compelling evidence for the reestablishment of *P. falciparum* sensitivity to CQ in the parasite population of Lambaréné, Gabon, and surrounding villages 21 years after its withdrawal from the national treatment guidelines. This is supported by a marked increase in the prevalence of wild-type alleles at *pfcrt*, the key molecular marker associated with CQ susceptibility, and a corresponding decrease in the median IC_50_s of CQ in ex vivo assays across parasite isolates collected over the study period. Moreover, the prevalence of *pfmdr1* 86 wild type has increased recently, reaching fixation in 2024 in the limited number of observed samples.

Chloroquine ex vivo analysis showed a progressive decrease of IC_50_s, with median values of 113.8 nM, 46.7 nM, 15.6 nM, and 2.1 nM in 2004, 2009, 2017–2018, and 2024, respectively. The stepwise increase in the wild-type *pfcrt* K76 and *pfmdr1* N86 alleles throughout the assessed period suggests a gradual replacement of drug-resistant parasite populations by CQ-susceptible strains. These molecular shifts are consistent with patterns observed in other endemic regions following CQ withdrawal, where the discontinuation of drug pressure removed the selective advantage previously conferred by resistant alleles, allowing wild-type genotypes, with higher fitness in the absence of CQ, to re-emerge and spread [22]. However, in Malawi [24], the reestablishment of the sensitive parasite population occurred more rapidly, with CQ-sensitive parasites detected in 2001, eight years after CQ replacement by sulfadoxine-pyrimethamine. In Gabon, CQ was officially replaced by ACTs in 2003, but in the area of this study, CQ treatment failures reported in the early 1990s shifted Albert Schweizer Hospital’s treatment policy already in this decade. Nevertheless, genotyping and drug-susceptibility studies repeatedly reported a prevalence of CQ-resistant parasites in the area, as high as 97–100% [34,35,36]. One could hypothesize that this delay in the reappearance of sensitive parasites is explained by using amodiaquine as a partner drug in ACTs, sustaining the drug pressure on the parasite population. Amodiaquine and CQ are 4-aminoquinoline compounds sharing similar modes of action by interfering with heme detoxification in the parasite’s digestive vacuole, leading to toxic heme accumulation and parasite death. However, the *pfcrt* wild-type K76 still reemerged despite the continuous use of amodiaquine in combination with artesunate, so it is unlikely that amodiaquine exerts the same pressure on *pfcrt* as CQ. Alternatively, this observation may be attributed to other factors. The *pfmdr1* 86**Y** mutation contributes to the fitness of *pfcrt* mutant parasites, as previously described [42]; it would enhance the fitness of *pfcrt*-mutant parasites by modulating the function of transporters and optimizing drug efflux [43]. Our data are in accordance with this, given that 76.9% of the isolates from 2009 harbored the mutant haplotype CV**IET** 86**Y**, and in the most recent population, 100% of the genotyped samples at both loci harbored the wild-type alleles CVMNK N86. Previous studies showed that wild-type *pfcrt* K76 and *pfmdr1* N86 are selected by artemether-lumefantrine treatment [44], which is another ACT in use in Gabon [1]. Therefore, it is plausible that this combination has contributed to the selection of wild-type alleles.

The high prevalence of *pfcrt* K76 and *pfmdr1* N86 observed in isolates from 2024 is of importance, given that they were associated with reduced lumefantrine susceptibility in other studies [45,46,47]. Data from 5000 clinical trial participants showed that the *pfmdr1* N86 allele was a significant risk factor for recrudescence following treatment with artemether-lumefantrine [46]. Temporal analysis of *P. falciparum* ex vivo susceptibility to lumefantrine showed an increase in the IC_50_s throughout the years in some African countries, although the drug has remained active at low nM concentrations [48,49]. Therefore, surveillance of the current lumefantrine response in the area of this study is of importance, due to its potential clinical relevance.

The frequency of the *pfcrt* **S**VMN**T** haplotype, linked to amodiaquine resistance in South America and in East African countries [14], was minimal in our dataset. This is in accordance with previous studies showing that the prevalence of **S**VMN**T** in Central and West Africa is low or even absent [50,51]. A comprehensive study, with samples collected between 2015 and 2018 across seven West and Central African countries, reported no occurrence of the **S**VMN**T** haplotype in the assessed samples [52]. This molecular profile suggests that the parasite population in these countries remains susceptible to amodiaquine. This could support a preference for the use of artesunate-amodiaquine combination even while artemether-lumefantrine efficacy remains above the WHO’s 10% failure threshold for treatment replacement.

Regionally, countries neighboring Gabon show varying trends following the cessation of CQ use. Whereas Equatorial Guinea presented a steady decline in the prevalence of mutant parasites [53,54,55,56], as in Gabon, regional differences were observed in Cameroon. A higher prevalence of resistant genotypes in Northern Cameroon compared to the coastal region could be explained by lower transmission in the North that would allow resistant strains to persist [57]. In the Republic of the Congo, in the prevalence of the *pfcrt* 76**T** mutant allele increased in 2022 compared to the previous year [58]. This discrepancy could stem from the most recent cohort consisting solely of people living with HIV, whose altered immunity can shift parasite dynamics [59].

Among study limitations, the lack of in vivo and genotyping paired data may preclude direct genotype–phenotype association analysis. Also, ex vivo and genotyping paired data from 2024 were obtained for a small sample size. The convenience sampling used here results in a heterogeneous population with varying baseline characteristics, which can introduce biases, e.g., the presence of symptoms [60].

Although a reversal of CQ resistance has been observed, its reintroduction, either in combination therapies or for specific cases, such as intermittent preventive treatment in pregnancy or seasonal malaria chemoprevention, should be considered only as a last choice. CQ reintroduction could reestablish drug pressure and rapidly reselect resistant parasites present in the parasite population at low frequencies or in neighboring countries. Luckily, there are currently several drugs in the pipeline that could replace current drug regimens in case of resistance. However, if these options fail, chloroquine or improved derivatives can be a valuable option.

Ultimately, monitoring parasite drug response and resistance genetic markers remains essential, as it provides valuable insights into the evolutionary trajectory of resistance alleles, helps detect potential cross-resistance, and informs future treatment strategies. As for the two genes assessed here, both remain paramount for managing current antimalarial policies, as they modulate sensitivities to the partner drugs lumefantrine and amodiaquine used in ACTs.

## 4. Methods and Materials

### 4.1. Study Site and Samples

Samples from seven studies conducted in Lambaréné, Gabon, and surrounding villages between 2004 and 2024 were included (Table 3). Lambaréné is located within the equatorial rainforest and is highly endemic for malaria. The transmission is perennial, and standard malaria treatment includes the ACTs artemether-lumefantrine and artesunate-amodiaquine. Detailed information on the inclusion criteria, sample processing, and the number of samples per study is listed in Table 3 and Table 4.

Studies 1 and 2 were approved by the ethics committee of the International Foundation for the Albert Schweitzer Hospital in Lambaréné. The Institutional Ethics Committee of the Centre de Recherches Médicales de Lambaréné (CERMEL) approved the other studies under the respective reference numbers: study 3 (CEI-007/2014), study 4 (CEI-CERMEL015/2015), study 5 (CEI/CERMEL006/2019), study 6 (CEI-020/2018), and study 7 (CEI-012/2023). Informed consent was provided by adult participants and parents or guardians of children and adolescents under 18 years of age.

### 4.2. Clinical Isolate Collection and Processing for Ex Vivo Assessment of Chloroquine Response

*P. falciparum* clinical isolates were collected from individuals according to the inclusion criteria in Table 3 (studies 1, 2, 4, and 7). Participants reporting use of antimalarial drugs in the previous 30 days were excluded. Asymptomatic and symptomatic individuals were screened for *P. falciparum* infection by microscopy (studies 1 and 2) or by rapid diagnostic test (studies 4 and 7); parasitemia was confirmed by Giemsa-stained thick blood smear. Individuals with *P. falciparum* monoinfection ≥ 1000 parasites/μL, as determined by microscopy, and willing to participate were enrolled, and venous blood was collected in vacutainer tubes. Samples were immediately processed. Blood was centrifuged; plasma and buffy coat were removed, and the parasitemia adjusted to 0.02% (study 1) or 0.05% (other ex vivo studies).

Next, 96-well plates were precoated with CQ in a 3-fold dilution, and the clinical isolates were added in duplicate to complete culture medium at 1.5% hematocrit. After 3 days of incubation at 37 °C in a candle jar, plates were frozen until analyzed by Histidine Rich Protein 2–ELISA [39,62]. Parasite growth inhibition was measured by spectrophotometry.

### 4.3. Assessment of Molecular Markers Associated with Chloroquine Response

#### 4.3.1. DNA Extraction

Samples from studies 2, 3, 4, 5, 6, and 7 were assessed for molecular markers associated with CQ response. No samples were available for retrospective molecular analysis from ex vivo study 1. DNA from whole blood was extracted with commercial kits following the manufacturer’s instructions. Some samples (studies 4, 5, and 6) were preserved in RNAlater, which was removed, and PBS 1× was added to the pellet prior to extraction with the kit. Extraction details are found in Table 3. While samples from study 2 were collected in 2009, DNA integrity was preserved by storage in Tris-EDTA buffer at a constant −20 °C. Multiple freeze-thaw cycles were avoided. The reliability of the template was confirmed by successful real-time PCR, conventional PCR, and high-quality Sanger sequencing.

#### 4.3.2. *Plasmodium* spp. Detection and Quantification

*Plasmodium* was detected and quantified via RT-qPCR targeting *18S* rRNA and DNA [63]. *Plasmodium* species were identified by qPCR with a preamplification step, also targeting the *18S* rRNA gene. Primers and probes were used according to published protocols [37,64]. Assays were performed on a LightCycler 480 Instrument II (Roche, Basel, Switzerland).

#### 4.3.3. *pfcrt* Genotyping

The *pfcrt* gene was genotyped using primers and probes [38,65] to detect the *pfcrt* haplotypes CVMNK, **S**VMN**T**, and CV**IET** at codons 72–76. Samples were preamplified via conventional PCR, except for studies 4 and 5. Samples from studies 4 and 5 that were negative in the first run were repeated, including a preamplification step.

Samples were run in duplicate. DNA from *P. falciparum* strains 3D7 and D10 were used as positive controls for the sensitive CVMNK haplotype, and Dd2 and 7G8 for the resistant CV**IET** and **S**VMN**T** haplotypes, respectively. DNA from the whole blood of a malaria-naïve person and nuclease-free water were included as non-template controls. Reactions were performed on a LightCycler 480 Instrument II (Roche, Basel, Switzerland). The quantification cycle values were calculated with the second-derivative maximum method (LightCycler 480 software, version 1.5.1.62) after color compensation.

#### 4.3.4. *pfmdr1* Genotyping

Two fragments of the *pfmdr1* gene, comprising codons 86, 184, 1034, 1042, and 1246, were amplified by nested PCR [66] followed by Sanger sequencing. Amplicons were visualized on the QIAxcel System, purified with ExoSAP It (ThermoFisher, Waltham, MA, USA), and sent for Sanger sequencing to Eurofins Genomics. Sequences were examined in the Geneious program version 2025.2.1 against the *P. falciparum* 3D7 reference sequence of *pfmdr1* (PF3D7_0709000).

The detailed PCR conditions, primers, and probe sequences used in this study are described in Table 5.

### 4.4. Statistics

Individual inhibitory concentrations (IC_50_) were determined by non-linear regression analysis of log-concentration-response. Data for the clinical isolates were presented using the median IC_50_ and interquartile range (IQR). Due to a significant discrepancy from the expected trend, IC_50_ data from one ex vivo isolate from 2024 were deemed an outlier and removed from the analysis.

IC_50_ values variations among the periods of study and *pfcrt* haplotypes were assessed using the non-parametric Kruskal-Wallis H test. Dunn’s Multiple Comparisons test was performed for post-hoc analyses, using the dunn.test package of R version 4.3.2. Pearson’s Chi-squared test was used to compare the percentages of *pfcrt* CVMNK haplotype throughout the assessed years. IC_50_ data were log transformed, and multiple regression was performed to assess the association of *pfmdr1* SNPs with IC_50_s in R (version 4.3.2).

## 5. Conclusions

This study provides evidence of the reestablishment of *P. falciparum* sensitivity to CQ in Lambaréné, Gabon, and neighboring areas, two decades after its withdrawal from national malaria treatment guidelines. The steady increase in wild-type *pfcrt* and *pfmdr1* alleles, alongside the decrease in CQ IC_50_ values, supports a gradual replacement of resistant parasite populations by sensitive strains. Our findings highlight that a comprehensive analysis of *P. falciparum* susceptibility to the main antimalarial drugs, coupled with investigation of molecular markers, can advance our understanding of antimalarial resistance and assist in the optimization of therapeutic strategies. By integrating drug susceptibility testing with molecular profiling, we can identify emerging resistance patterns and elucidate the underlying mechanisms of drug failure. Continuous surveillance is essential to stay ahead of evolving resistance and to ensure that existing and future antimalarial drugs remain effective in the fight against malaria.

## Figures and Tables

**Table 1:** Distribution of *pfcrt* haplotypes (codons 72–76) in *Plasmodium falciparum* isolates collected from 2009 to 2024 in Lambaréné and surrounding villages in Gabon. Mutant amino acids are underlined. Data from 2016 have been previously published; data from other time points were generated in this study.

Year	*N*	*pfcrt* Haplotype *n* (%)						
		Mono Infections			Mixed Infections			
		CVMNK	CVIET	SVMNT	CVMNK/CVIET	CVMNK/SVMNT	CVIET/SVMNT	CVMNK/CVIET/SVMNT
2009	28	1 (3.6)	25 (89.3)	1 (3.6)	0 (0)	1 (3.6)	0 (0)	0 (0)
2016 *	335	38 (11.3)	223 (66.6)	1 (0.3)	70 (20.9)	2 (0.6)	0 (0)	1 (0.3)
2017–2018	46	16 (34.8)	22 (47.8)	0 (0)	8 (17.4)	0 (0)	0 (0)	0 (0)
2019–2020	86	52 (60.5)	18 (20.9)	0 (0)	16 (18.6)	0 (0)	0 (0)	0 (0)
2019–2021	145	85 (58.6)	38 (26.2)	2 (1.4)	18 (12.4)	1 (0.7)	1 (0.7)	0 (0)
2024	57	56 (98.2)	0 (0)	0 (0)	0 (0)	1 (1.8)	0 (0)	0 (0)

* Data from 2016 has been previously published [37].

**Table 2:** Distribution of IC_50_ values and *pfmdr1* haplotypes and *pfcrt* haplotypes in combination with *pfmdr1* codon 86 in *Plasmodium falciparum* isolates collected from 2009 to 2024 in Lambaréné and surrounding villages in Gabon. Mutant amino acids are bold and underlined. Isolates identified with mixed genotypes are considered mutants.

Gene and Codons	Haplotypes	2009 % (*n*/*N*)	2017–2018 % (*n*/*N*)	2024 % (*n*/*N*)	Chloroquine Median IC_50_, IQR (nM)
*pfmdr1* 86, 184, 1034, 1042, 1246	N_86_Y_184_S_1034_N_1042_D_1246_	23.1 (6/26)	37.2 (16/43)	47.4 (9/19)	na
	N**F**SND	0 (0/26)	44.2 (19/43)	52.6 (10/19)	na
	**YF**SND	76.9 (20/26)	16.3 (7/43)	0 (0/19)	na
	**Y**YSN**Y**	0 (0/26)	2.3 (1/43)	0 (0/19)	na
*pfcrt* 72–76 and *pfmdr1* 86	C_72_V_73_M_74_N_75_K_76_ N_86_	3.8 (1/26)	27.9 (12/43)	100 (18/18)	3.97, 0.85–7.86
	CVMNK**Y**	0 (0/26)	4.7 (2/43)	0 (0/18)	*
	CV**IET** N	15.4 (4/26)	58.1 (25/43)	0 (0/18)	34.2, 18.4–58.0
	CV**IET Y**	76.9 (20/26)	9.3 (4/43)	0 (0/18)	46.2, 29.3–81.2
	**S**VMN**T** N	3.8 (1/26)	0 (0/43)	0 (0/18)	*

na = not assessed, * = median and IQR not calculated due to small sample size.

**Table 3:** Overview of the seven studies included.

Study Number	Sampling Period	Sampling Area	Age	Clinical Status	Parasitemia	No. Included Samples	Blood Storage	DNA Extraction	Pan *Plasmodium* qPCR	*Plasmodium* Species qPCR	*pfcrt* Data Published	Reference	Data
					(Parasites/µL)								
1	August–November 2004	Lambaréné	NA	Uncomplicated malaria	1000–120,000	42	Not done				No	[41]	Ex vivo
2	February–May 2009	Lambaréné	NA	Uncomplicated malaria	NA	28	According to [37]				No	[39]	Ex vivo + genotyping
3	February/March 2016	rural areas of Gabon (Fougamou and villages in surroundings)	1–96 y	Asymptomatic and symptomatic	NA	335	According to [37]				Yes [37]	[37]	genotyping
4	October 2017–June 2018	Lambaréné and surrounding villages	1–5 y or ≥18 y	NA	≥1000	48	500 µL whole blood + 1.3 mL RNAlater (ThermoFisher Scientific, Waltham, MA, USA)	automated (QIAsymphony, Qiagen, Hilden, Germany)	According to [37]		No	[40]	Ex vivo + genotyping
5	June 2019–October 2020	Lambaréné and surrounding villages	≥18 y	Asymptomatic	200–5000	86	500 µL whole blood + 1.3 mL RNAlater (ThermoFisher Scientific, Waltham, MA, USA)	QIAamp Blood Mini Kit (Qiagen, Hilden, Germany) or automated in the KingFisher Flex System with sbeadex Blood DNA Purification Kit (LGC, Teddington, UK)	According to [61]		No	[61]	genotyping
6	January 2019–August 2021	Lambaréné and surrounding villages	≥12 m	Asymptomatic and symptomatic	1–355,625 *	154	500 µL whole blood in EDTA tube + 1.3 mL RNAlater (ThermoFisher Scientific, Waltham, MA, USA)	QIAamp Blood Mini Kit (Qiagen, Hilden, Germany)	According to [61]	According to [61], with the following modification: 1.3 mM MgCl_2_ added in the preamplification master mix.	No	Not published	genotyping
7	January–August 2024	Lambaréné and surrounding villages	>2 y	Asymptomatic and symptomatic	≥1000	57	Whole blood in EDTA/citrate tube and cell culture pellet in glycerolyte	QIAamp Blood Mini Kit (Qiagen, Hilden, Germany)	Not done	Not done	No	Not published	Ex vivo + genotyping

* Calculated by qPCR; y = years; m = months; NA = not assessed.

**Table 4:** Sample numbers from the ex vivo and genotyping assays.

Study Year	*N* [Positive Ex Vivo Assay]	*N* [Positive *pfcrt* Genotyping]	N [Positive *pfmdr1* Genotyping]	*N* [Positive Paired Ex Vivo and *pfcrt* Data]	*N* [Positive Paired Ex Vivo and *pfmdr1* Data]
2004	42	NA	NA	NA	NA
2009	26	28	26	26	26
2016	NA	335	NA	NA	NA
2017–2018	46	46	43	44	43
2019–2020	NA	86	NA	NA	NA
2019–2021	NA	145	NA	NA	NA
2024	39	57	19	18	18

NA = not analyzed.

**Table 5:** Oligo sequences and PCR conditions used in the study.

Target Gene	PCR Assay	Oligo Name	Oligo Sequence (5′-3′)	PCR Conditions	Cycling Conditions	Reference
*18S* rRNA	Pan *Plasmodium* RT-qPCR	PLU3 Fwd	GCTCTTTCTTGATTTCTTGGATG	1.5 µL of total nucleic acids template, 1× TaqMan^TM^ RNA-to-CT^TM^ 1-step Kit (Thermofisher Scientific, Waltham, MA, USA), 1× TaqMan RT-PCR Enzyme Mix (Thermofisher Scientific, Waltham, MA, USA), 0.4 µM forward and reverse primer each, 0.15 µM probe. Total reaction volume 10 µL	48 °C/20 min; 96 °C/10 min; [95 °C/15 s, 62 °C/60 s] × 45	[63,67]
		PLU3 Rev	AGCAGGTTAAGATCTCGTTCG			
		PLU3 Probe	HEX-ATGGCCGTTTTTAGTTCGTG-MGBEQ			
	Pan *Plasmodium* preamplification	rPLU5	TTAAAATTGTTGCAGTTAAAACG	5 µL of DNA template, 1× Buffer (Qiagen, Hilden, Germany), 0.25 mM dNTPs, 1 U Taq DNA Polymerase (Qiagen, Hilden, Germany) and 0.5 µM forward and reverse primer each. Total reaction volume 50 µL	95 °C/5 min; [95 °C/30 s, 58 °C/30 s, 72 °C/1 min 20 s] × 20; 72 °C/5 min	[68]
		rPLU6	CCTGTTGTTGCCTTAAACTTC			
	*Plasmodium* spp. qPCR	Pf-1	ATTGCTTTTGAGAGGTTTTGTTACTTT	2.5 µL of pre-amplified template, 1× SensiFAST^TM^ Probe No-ROX Kit (Meridian Bioscience, Cincinnati, OH, USA), 0.4 µM forward and reverse primers each, 0.15 µM probe. Total reaction volume 10 µL	95 °C/3 min; [95 °C/10 s, 60 °C/30 s] × 45	[69]
		Pf-2	GCTGTAGTATTCAAACACAATGAACTCAA			
		Pf-Probe	HEX-CATAACAGACGGGTAGTCAT MGBEQ			
		Pm-TFwd	GGTGTTGGATGATAGAGTAA			
		Pm-TRev	CCCAAAGACTTTGATTTCTC			
		Pm-TProbe	HEX-AGGAAGCTATCTAAAAGAAACACTCAT-BHQ1			
		PO-18S-S-Fwd	ATTTCAAAGAGTCATGGCGTTTCTG			
		POC-18S-S-Rev	TTGTAAAGGAGACACTTTCTTGAAATCG			
		POC-18S-S-Probe	FAM-CTCCTTGGTCGATCTGCCCAGCACT-BHQ1			
		POW-18S-S-Rev	TGTAAAGGAGACAACTTTCTTGGAGCTA			
		POW-18S-S-Probe	HEX-TTGATCGCCCAGCACTGACCATCT-BHQ1			
*pfcrt*	*pfcrt* preamplification	Pfcrt-Fwd	TGGCTCACGTTTAGGTGGAGGTTCTTG	2.5 µL of DNA template, 1× Buffer (Qiagen, Hilden, Germany), 0.1 mM dNTPs, 1 U Taq DNA Polymerase (Qiagen, Hilden, Germany) and 0.4 µM forward and reverse primer each. Total reaction volume 20 µL	94 °C/5 min; [94 °C/30 s, 55 °C/30 s, 72 °C/30 s] × 20; 72 °C/5 min	[38]
		Pfcrt-Rev	ACTGAACAGGCATCTAACATGGATATAGC			
	*pfcrt* qPCR	Pfcrt-Fwd	TGGTAAATGTGCTCATGTGTTT	2.0 µL of pre-amplified template, 1× SensiFAST^TM^ Probe No-ROX Kit (Meridian Bioscience, Cincinnati, OH, USA), 0.25 µM each probe, 0.4 µM forward and reverse primer each. Total reaction volume 10 µL	96 °C/10 min; [95 °C/15 s, 55 °C/60 s] × 40	[65]
		Pfcrt-Rev	AGTTTCGGATGTTACAAAACTATAGT			
		CVMNK Probe	FAM-TGTGTAATGAATAAAATTTTTGCTAA-BHQ1			
		CVIET Probe	HEX-TGTGTAATTGAAACAATTTTTGCTAA-BHQ1			
		SVMNT Probe	CY5-AGTGTAATGAATACAATTTTTGCTAA-BHQ2			
*pfmdr1*	*pfmdr1* nested PCR	Pfmdr1_2N1_Foward	AATTTGATAGAAAAAGCTATTGATTATAA	1.5 µL of DNA/pre-amplified template, 1× Buffer (Qiagen, Hilden, Germany), 1.5 mM MgCl_2_, 0.2 mM dNTPs, 1 U Taq DNA Polymerase (Qiagen, Hilden, Germany), 0.5 µM forward and reverse primer each. Total reaction volume 25 µL	95 °C/3 min; [95 °C/30 s, 52 °C/30 s, 72 °C/60 s] × 30; 72 °C/5 min	[66]
		Pfmdr1_2N1_Reverse	TATTTGGTAATGATTCGATAAATTCATC			
		Pfmdr1_2N2_Foward	GAATTATTGTAAATGCAGCTTTA			
		Pfmdr1_2N2_Reverse	GCAGCAAACTTACTAACACG			

HEX = 6-hexachlorofluorescein. MGBEQ = minor groove binder, eclipse quencher. BHQ-1/2 = black hole quencher-1/2. FAM = 6-carboxyfluorescein. CY5 = Cyanine 5.

## Data Availability

The original contributions presented in this study are included in the article/Supplementary Materials. Further inquiries can be directed to the corresponding author.

## Supplementary Materials

The following supporting information can be downloaded at: https://www.mdpi.com/article/10.3390/ijms27083566/s1.
