# Supplementary material for: Reversal of Chloroquine Resistance in Plasmodium falciparum in Gabon: A Phenotype-Genotype Relationship over the Last 20 Years
Source: Int J Mol Sci. 2026 Apr 16;27(8):3566. doi: 10.3390/ijms27083566 (PMC13116125; doi:10.3390/ijms27083566)
Supplement: Supplementary file 1 [file ijms-27-03566-s001.zip › ijms-4237252-supplementary.pdf]

Table S1: Articles on prevalence of *pfcrt* 76**T** polymorphism in countries neighboring Gabon.

Country and year (s)	PMID	Authors	Title
Cameroon, 2003, 2012	28347314	Ndam et al.	Reemergence of chloroquine-sensitive pfcrt K76 Plasmodium falciparum genotype in southeastern Cameroon
Cameroon, 2013	29110722	Apinjoh et al.	Molecular markers for artemisinin and partner drug resistance in natural Plasmodium falciparum populations following increased insecticide treated net coverage along the slope of mount Cameroon: cross-sectional study
Cameroon, 2004, 2014, 2019	37028468	Niba et al.	Evolution of Plasmodium falciparum antimalarial drug resistance markers post-adoption of artemisinin-based combination therapies in Yaounde, Cameroon
Cameroon, 2019	40635523	Kojom Foko et al.	Genetic epidemiology of Plasmodium falciparum chloroquine resistance in coastal, North and Far North areas of Cameroon
Republic of the Congo, 2009	22463364	Koukouikila-Koussounda et al.	Genetic polymorphism of merozoite surface protein 2 and prevalence of K76T pfcrt mutation in Plasmodium falciparum field isolates from Congolese children with asymptomatic infections
Republic of the Congo, 2010, 2014	28420403	Koukouikila-Koussounda et al.	Molecular surveillance of Plasmodium falciparum drug resistance in the Republic of Congo: four and nine years after the introduction of artemisinin-based combination therapy
Republic of the Congo, 2021	39543235	Baina et al.	Polymorphisms in the Pfcrt, Pfmdr1, and Pfk13 genes of Plasmodium falciparum isolates from southern Brazzaville, Republic of Congo
Republic of the Congo, 2022	41444531	Djontu et al.	Characterization of Plasmodium falciparum infections among people living with HIV under antiretroviral and co-trimoxazole chemoprophylaxis in Brazzaville, Republic of Congo
Equatorial Guinea, 2013	28086777	Berzosa et al.	Profile of molecular mutations in pfdhfr, pfdhps, pfmdr1, and pfcrt genes of Plasmodium falciparum related to resistance to different anti-malarial drugs in the Bata District (Equatorial Guinea)
Equatorial Guinea, 2011	26325683	Li et al.	Molecular mutation profile of Pfcrt and Pfmdr1 in Plasmodium falciparum isolates from Bioko Island, Equatorial Guinea
Equatorial Guinea, 2017	35670601	Liu et al.	Molecular Surveillance of Artemisinin-Based Combination Therapies Resistance in Plasmodium falciparum Parasites from Bioko Island, Equatorial Guinea
Equatorial Guinea, 2004, 2006, 2011, 2013, 2016, 2019	34906159	Berzosa et al.	Temporal evolution of the resistance genotypes of Plasmodium falciparum in isolates from Equatorial Guinea during 20 years (1999 to 2019)
Equatorial Guinea, 2018	34505447	He et al.	Drug-resistant gene polymorphisms in Plasmodium falciparum isolated from Bioko Island, Equatorial Guinea in 2018 and 2019
Gabon, 2016	31278305	Woldearegai et al.	Characterization of Plasmodium infections among inhabitants of rural areas in Gabon
